# Haplotypes of [‐794(CATT)_5–8_/‐173G>C] *MIF* gene polymorphisms and its soluble levels in cutaneous squamous cell carcinoma in western Mexican population

**DOI:** 10.1002/mgg3.2252

**Published:** 2023-07-24

**Authors:** Elizabeth Guevara‐Gutiérrez, Marina Ramos‐Súarez, Romina Angélica Villalobos‐Ayala, Alberto Tlacuilo‐Parra, José Francisco Muñoz‐Valle, Victor Tarango‐Martínez, Yeminia Valle, Jorge Ramón Padilla‐Gutiérrez, José Manuel Rojas‐Díaz, Emmanuel Valdés‐Alvarado

**Affiliations:** ^1^ Departamento de Dermatología, Instituto Dermatológico de Jalisco "Dr. José Barba Rubio" Secretaría de Salud Jalisco Zapopan Mexico; ^2^ División de Investigación, Unidad Médica de Alta Especialidad (UMAE), Hospital de Pediatría Centro Médico Nacional de Occidente, IMSS Guadalajara Mexico; ^3^ Instituto de Investigación en Ciencias Biomédicas, Centro Universitario de Ciencias de la Salud Universidad de Guadalajara Guadalajara Mexico

**Keywords:** carcinoma, squamous cell, cytokines, haplotypes, macrophage migration inhibition factors, polymorphism, genetic

## Abstract

**Background:**

Some cytokines are strongly implicated in the development of squamous cell carcinoma (SCC) such as the Macrophage migration inhibitory factor (MIF). The haplotype ‐794 (CATT)_5–8_/‐173G>C in *MIF* gene polymorphisms has been associated with some types of cancer. The aim of this study is to establish the possible association between the presence of this haplotype in the *MIF* gene and its subsequent soluble levels with the susceptibility of SCC in western Mexican population.

**Methods:**

This study included 175 SCC patients and 175 age–sex‐matched individuals as a reference group (RG) from western Mexico. Genomic DNA was extracted from peripheral blood leukocytes. Polymorphisms were genotyped by endpoint PCR and PCR‐RFLP, and the determination of MIF serum levels was measured by ELISA. Clinical characteristics were evaluated by a group of dermatologists.

**Results:**

Analysis of [‐794(CATT)_5–8_/‐173G>C] *MIF* gene polymorphisms showed that the 5C (OR = 2.7, *p* = 0.02) and the 7G (OR = 3.39, *p* < 0.01) haplotypes are associated with susceptibility in SCC. MIF soluble levels in SCC patients showed a median of 13.93 ng/mL, whereas the reference group showed 6.000 ng/mL.

**Conclusions:**

Our findings suggest that 5C and 7G [‐794(CATT)_5–8_/‐173G>C] *MIF* gene haplotypes are associated with susceptibility to SCC and that SCC patients present increased soluble levels of MIF.

## Graphical abstract

- MIF gene polymorphisms may lead to an overexpression of its protein.
- Haplotypes in the MIF gene may be associated with a risk factor in squamous cell carcinoma.

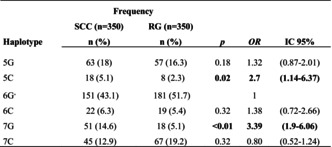

## 1 INTRODUCTION

Nonmelanoma skin cancers (NMSC) are the most common types of neoplasms, and their incidence is increasing worldwide. The two major subtypes of these dermatological cancers are basal cell carcinoma (BCC) and squamous cell carcinoma (SCC) (Lomas et al., 2012). In concordance with world epidemiological data, in Mexico, NMSCs are very common, and notwithstanding, there is a poor register practice in Mexico of these neoplasms, and the incidence of cases seems to be higher every year (Jurado‐Santa Cruz et al., 2011). SCC is the second most common type of NMSC, and it is estimated that approximately 14%–20% of the population will have it at some point in their lives. This cancer is particularly important due to its metastasis risk, especially compared with other NMSCs which may be up to 30% (Waldman & Schmults, 2019). SCC is a multifactorial disease like all types of cancer, but some factors may increase the risk of developing these neoplasms. UV radiation exposure is considered the major risk factor in the carcinogenesis of skin cancers, but some other factors can play a significant role, such as X‐rays, arsenic, or carcinogenic chemicals exposure, and even HPV infection (Didona et al., 2018). Cytokines are strongly implicated in the development of cancer within chronically inflamed tissues. Certain cytokines promote the development of a pro‐tumorigenic microenvironment by orchestrating the recruitment of immune cells which leads to promoting cancer development and progression (Gordon‐Weeks et al., 2015). Macrophage migration inhibitory factor (MIF; OMIM accession number: 153620) is a pluripotent cytokine involved in a wide range of pathophysiologic events in association with inflammation, innate and acquired immune responses, and cell growth (Kang & Bucala, 2019). Several reports have suggested that MIF plays a critical role in tumorigenesis (Bach et al., 2008), angiogenesis (Amin et al., 2003), and metastasis (Sumaiya et al., 2022). According to this, overexpression of MIF has been found in several human neoplasms, including prostate (Wang, Yao, et al., 2019), breast (Avalos‐Navarro et al., 2019), colorectal (Olsson et al., 2020), endometrial (Xiao et al., 2016), hepatocellular (Zhao et al., 2011), and pancreatic ductal cancer cells (Denz et al., 2010), as well as NMSC (Martin et al., 2009). Two functional polymorphisms have been located in the promoter region of the *MIF* gene, the first polymorphism is a CATT short‐tandem repeat (STR) at position ‐794, with five‐ to eight‐length variants (alleles 5–8), in which the number of repeats of CATT is associated with varying amounts of serum circulating levels of MIF (Donn et al., 2002). In fact, the variants with the higher repetitions (CATT)_6–8_ show higher circulating MIF levels (Avalos‐Navarro et al., 2020). The second promoter polymorphism is a single nucleotide polymorphism (SNP) in the position ‐173 and consists of a transversion of G>C which has been associated with increased *MIF* gene expression and protein levels (Llamas‐Covarrubias et al., 2013). Although there are a few investigations that link the haplotype ‐794 (CATT)_5–8_/‐173G>C in some types of cancer, it has never been found the correlation between SCC with these genetic variants in a Mexican population. For that reason, this study aims to establish if there is an association between the presence of this haplotype in the *MIF* gene and MIF soluble levels with the susceptibility of SCC carcinogenesis in the western Mexican population.

## 2 MATERIALS AND METHODS

### 2.1 Ethical compliance

The study was performed according to the ethical principles for experiments involving humans stated in the Declaration of Helsinki and ethical approval was obtained by Dirección General de Salud Publica (33/IDJ‐JAL/2016). Informed consent was obtained from all patients for being included in the study. Furthermore, submitting authors are responsible for coauthors declaring their interests.

### 2.2 Subjects

The study group included 350 Mexican mestizo subjects: 175 SCC histologically confirmed and unrelated patients were recruited from the “Instituto Dermatológico de Jalisco José Barba Rubio” in Guadalajara City, Mexico. 175 unrelated individuals identified as reference group (RG) age–sex matched with SCC patients. We considered Mexican mestizo subjects, only those individuals who for three generations, including their own, had been born in western Mexico.

### 2.3 Genotyping of *MIF* ‐794(CATT)_5–8_ and ‐173G>C polymorphisms

Genomic DNA was extracted from peripheral blood leukocytes using Miller's Technique (Miller et al., 1988). Genotyping of the polymorphisms in the *MIF* gene (NG_012099.1) was done as follows: STR ‐794(CATT)_5–8_ polymorphism was achieved by endpoint PCR (Forward primer: 5′‐TTG‐CAC‐CTA‐TCA‐GAG‐ACC‐3′ and Reverse primer: 5´‐TCC‐ACT‐AAT‐GGT‐AAA‐CTC‐G‐3′) with the following cycling conditions: initial denaturing 95°C for 4 min followed by 30 cycles of 30 s at 95°C, 30 s at 60°C, 30 s at 72°C, and a final extension of 2 min at 72°C. Amplification products were further electrophoresed on a 19:1 7% polyacrylamide gel at 150 V for 15 h and stained with AgNO_3_. 208, 212, 216, and 220 fragments were identified corresponding to alleles 5, 6, 7, and 8, respectively.

The ‐173G>C *MIF* polymorphism was genotyped by PCR‐RFLP (Forward primer: 5’‐ACT‐AAG‐AAA‐GAC CCG‐AGG‐C‐3′ and Reverse primer: 5′‐GGG‐GCA‐CGT‐TGG‐TGT‐TTA‐C‐3′). Cycling conditions: initial denaturing at 95°C for 4 min followed by 35 cycles of 30 s at 95°C, 30 s at 60°C, 30 s at 72°C, and a final extension of 2 min at 72°C. Amplification products (366 bp) were digested with *Alu I* restriction endonuclease (New England Biolabs, Ipswich, MA) by overnight incubation at 37°C. Amplification products were further electrophoresed on a 29:1 6% polyacrylamide gel at 150 V for 15 h and stained with AgNO_3._ The G allele resulted in 268 bp and 98 bp fragments, whereas the C allele was represented by 206 bp, 98 bp, and 62 bp fragments.

### 2.4 MIF serum levels

The determination of MIF serum levels was performed by enzyme‐linked immunosorbent assay (ELISA) and the commercial Human MIF ELISA Kit (RayBio®, USA, catalog number: ELH‐MIF‐1), according to the manufacturer's instructions. MIF assay sensitivity was 6 pg/mL.

### 2.5 Statistical analysis

A case–control analysis was performed. The Hardy–Weinberg equilibrium test and genotype and allele frequencies were calculated by the chi‐square test or Fisher's exact test, when applicable. Odds ratios (OR) and 95% confidence intervals (95% CI) were calculated to test the probability that the genotype and allele frequencies were associated with SCC. Haplotype inference was performed using EMHAPFREQ software (Excoffier & Slatkin, 1995). Linkage disequilibrium was estimated through the Lewontin D′ measure (LD). Haplotypic frequencies were also compared through chi‐square and Fisher exact tests. MIF serum levels were compared among groups by Student's *t* and ANOVA tests for MIF and U Mann–Whitney. A *p* value of <0.05 was considered statistically significant. All the statistical analyses were done with the SPSS 20.0 software.

## 3 RESULTS

### 3.1 Demographic and clinical characteristics

The median age of SCC patients was 72 years old, while the reference group (RG) was 70. Gender distribution between SCC individuals was 58% male and 42% female. 61.1% of the neoplasms in SCC patients had a size minor than 2 cm, whereas 38.9% presented neoplasms bigger than 2 cm. 63% of patients presented lesions in head or neck, 15% in the core, 15% in upper extremities, and just 6% in lower extremities. The histopathological study revealed that 24% of neoplasms were in situ while no invasive tumors were found. 66% of lesions showed well‐differentiated cells, 9% were moderately differentiated, and just 1% of neoplasms were poorly differentiated. All clinical characteristics are shown in Table 1.

**TABLE 1:** Clinical and demographic characteristics.

Variable	SCC (*n* = 175)	RG (*n* = 175)
	*n* (%)	*n* (%)
Demographics		
Age (years)^1^	72 (38–92)	70 (40–88)
Gender		
Male	102 (58)	100 (57)
Female	73 (42)	75 (43)
Clinical characteristics		
Tumor size		
<2 cm	107 (61.1)	‐
>2 cm	68 (38.9)	‐
Tumor localization		
Head–neck	110 (63)	‐
Core	26 (15)	‐
Upper extremities	27 (15)	‐
Lower extremities	10 (6)	
Histopathology		
In situ	43 (24)	‐
Invasive	0 (0)	‐
Well differentiated	115 (66)	‐
Moderately differentiated	15 (9)	‐
Poorly differentiated	2 (1)	‐

^1^ Data show minimum and maximum.

### 3.2 Allele and genotypic frequencies of *MIF* ‐794(CATT)_5–8_ and ‐173G>C polymorphisms

There was no deviation from the Hardy–Weinberg equilibrium for any of the polymorphisms in both groups (*p* > 0.05). Analysis of *MIF* ‐794(CATT)_5–8_ polymorphism showed that the most common genotype in both SCC and RG individuals was the heterozygote 5,6 (34.3% in SCC and 30.9% in RG). Allele 6 was the most frequent in both groups (49.7% in SCC and 56.9% in RG). A significant difference was found in the 6,6 genotype which may be considered a nonrisk factor (OR = 0.53, *p* = 0.03). Allele and genotypic frequencies of *MIF* ‐794(CATT)_5–8_ polymorphisms are shown in Table 2. Moreover, *MIF* ‐173G>C polymorphism analysis showed that the GG homozygote genotype was the most common in both study groups (52.6% for both) and the G allele was the most common (75.7 in SCC and 73.3 in RG). Nevertheless, genotype and allele frequencies for the ‐173G>C *MIF* polymorphism did not show any significant differences. Allele and genotypic frequencies of *MIF* ‐173G>C polymorphism are shown in Table 3.

**TABLE 2:** Allele and genotypic frequencies of *MIF* ‐794 (CATT)_5–8_ polymorphisms.

Polymorphism	SCC	RG	OR (IC 95%)	*p* values
	*n* = 175 (%)	*n* = 175(%)		
*MIF* ‐794 (CATT)_5–8_				
Genotype				
5,5	6 (3.4)	2 (1.1)	2.7(0.52–13.94)	*p* = 0.2
5,6	60 (34.3)	54 (30.9)	1	
5,7	9 (5.1)	8 (4.6)	1.01 (0.36–2.81)	*p* = 0.9
6,6	29 (16.6)	49 (28)	**0.53 (0.29–0.96)**	***p* = 0.03**
6,7^§^	56 (32)	47 (26.8)	1.07 (0.63–1.83)	*p* = 0.8
7,7	15 (8.6)	15 (8.6)	0.9 (0.40–2.01)	*p* = 0.8
Alelle				
5	81 (23.1)	66 (18.9)	1.28 (0.84–1.95)	*p* = 0.2
6^§^	174 (49.7)	199 (56.9)	1	
7	95 (27.2)	85 (24.2)	1.23 (0.84–1.80)	*p* = 0.28

Bold values are to highlight the statistical significance found.

^§^ Highlight the most common genotype in the Reference group.

**TABLE 3:** Allele and genotypic frequencies of *MIF* ‐173G>C polymorphisms.

Polymorphism	SCC	RG	*OR* (IC 95%)	*p* values
	*n* = 175 (%)	*n* = 175 (%)		
*MIF* ‐173G>C				
Genotype				
GG^§^	92 (52.6)	92 (52.6)	1	
GC	81 (46.3)	72 (41.1)	1.12(0.73–1.72)	*p* = 0.59
CC	2 (1.1)	11 (6.3)	**0.18 (0.04–0.84)**	***p* = 0.02**
Alelle				
G	265 (75.7)	256 (73.3)	1	
C^§^	85 (24.3)	94 (26.7)	0.87 (0.62–1.22)	*p* = 0.43

Bold values are to highlight the statistical significance found.

^§^ Highlight the most common genotype in the Reference group.

### 3.3 Haplotypes of [‐794(CATT)_5–8_/‐173G>C] *MIF* gene polymorphisms

The linkage disequilibrium analysis was performed and showed that the alleles of both polymorphisms are not segregated independently (D′ = 0.636). A comparison between haplotype distribution was made as shown in Table 4, and it showed that the most common haplotype in both groups was 6G (43.1% in SCC and 51.7% in RG). Two haplotypes had a significant association with SCC, those were the haplotypes 5C (OR = 2.7, *p* = 0.02) and 7G (OR = 3.39, *p* < 0.01).

**TABLE 4:** Haplotype frequencies of [‐794 (CATT)_5−8_/‐173 G>C] *MIF* gene polymorphisms in SCC and RG individuals.

Haplotype	Frequency		*p*	OR	IC 95%
	SCC (*n* = 350)	RG (*n* = 350)			
	*n* (%)	*n* (%)			
5G	63 (18)	57 (16.3)	0.18	1.32	(0.87–2.01)
5C	18 (5.1)	8 (2.3)	**0.02**	**2.7**	**(1.14–6.37)**
6G*	151 (43.1)	181 (51.7)		1	
6C	22 (6.3)	19 (5.4)	0.32	1.38	(0.72–2.66)
7G	51 (14.6)	18 (5.1)	**<0.01**	**3.39**	**(1.9–6.06)**
7C	45 (12.9)	67 (19.2)	0.32	0.80	(0.52–1.24)

Bold values are to highlight the statistical significance found.

* Highlight the most common genotype in the Reference group.

### 3.4 MIF soluble levels

**FIGURE 1:**
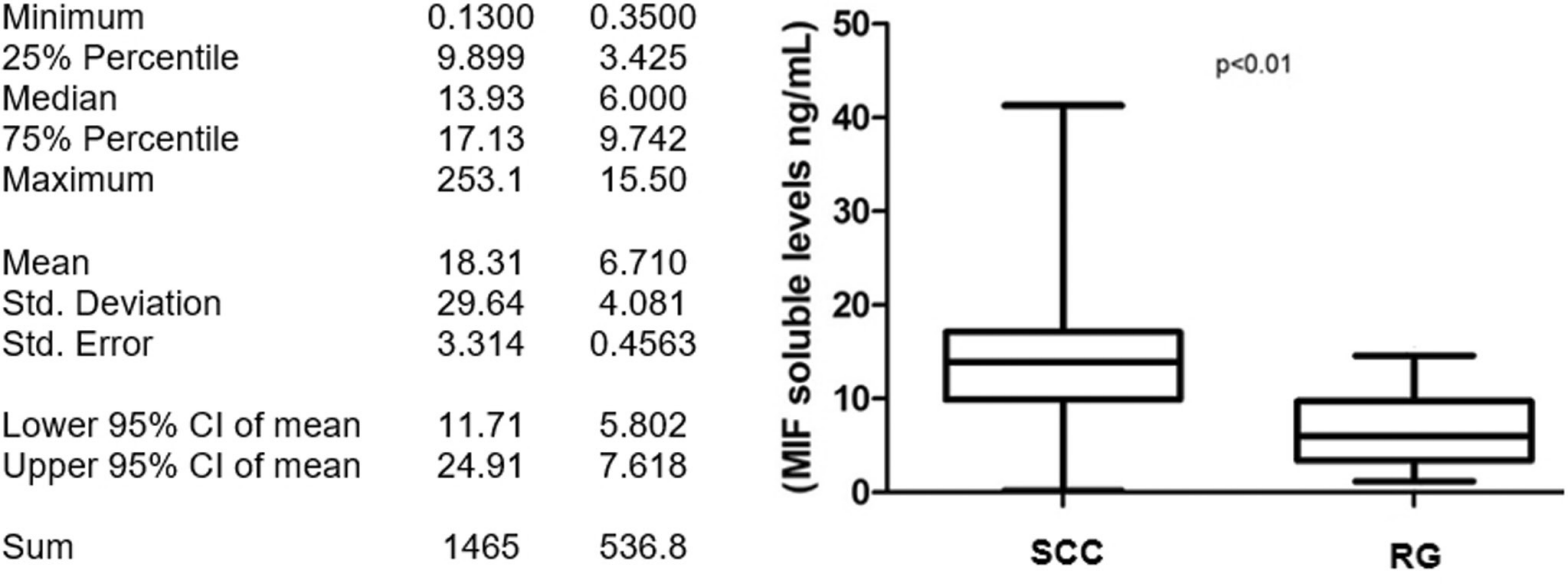
Soluble levels of MIF. MIF soluble levels are significantly increased in patients with SCC (13.93 ng/mL) compared with the reference group (6.000 ng/mL). comparison made by Student's t test with SPSS 20.0 software and considered a *p* value of <0.05 statistically significant.

MIF soluble levels were measured in SCC patients and RG individuals. The reference group had a median concentration of 6.00 ng/mL while SCC was more than twice this level, showing a median concentration of 13.93 ng/mL (*p* < 0.01). This data are shown in Figure 1.

## 4 DISCUSSION

SCC is the second most frequent dermatological neoplasm in the Mexican population, despite there being poor register practice in Mexico (Alfaro‐Sánchez et al., 2016). According to some studies that have described a higher incidence of SCC among men and in old individuals (Green & Olsen, 2017), we found a slightly increased incidence in men (58%) and a median age of SCC development at 72 years old. Moreover, we found that 93% of SCC neoplasms were located on sun‐exposed parts of the body (head–neck 63%, core 15%, and upper extremities 15%) which is consistent with the high exposure to sun rays and the population's low general use of sunscreen (Castanedo‐Cazares et al., 2006; Green & Olsen, 2017). Regardless chronic exposure to UV radiation is the most relevant factor that may lead to SCC carcinogenesis, cytokines like MIF as well as genetic variants have been for years linked to risk, development, and progression of NMSC such as SCC (Tsai & Tsao, 2004). MIF increases malignant transformation, tumor growth, and metastatic potential, moreover, in many tumor cells and pretumor states elevated MIF and mRNA levels have been observed (Xu et al., 2013).

The ‐794 (CATT)_7_ and ‐173*C alleles of *MIF* gen exhibit higher MIF circulating levels, and it is important to remark that there is a strong linkage disequilibrium among ‐794 (CATT)_7_ and ‐173*C alleles, which is also associated with a higher expression (Llamas‐Covarrubias et al., 2013). Our results show that in the ‐794(CATT)_5–8_ *MIF* polymorphism, the allele 6 is the most common in the western Mexico population, as was described in other diseases such as rheumatoid arthritis (Llamas‐Covarrubias et al., 2013), multiple sclerosis (Castañeda‐Moreno et al., 2018), and acute coronary syndrome (Valdés‐Alvarado et al., 2014). Furthermore, the 5,6 genotype was the most common and the 6,6 genotype seems to be associated with a lower‐risk factor (OR = 0.53, *p* = 0.03). It is important to highlight that short repetitions of this polymorphism (like the 6,6 genotype) are associated with a less MIF expression (Llamas‐Covarrubias et al., 2013) and this could have an impact on several pathologies such as multiple sclerosis (Castañeda‐Moreno et al., 2018) or basal cell carcinoma (Guevara‐Gutiérrez et al., 2021). Our results also showed that in accordance with other studies, in western Mexico ‐173 *MIF* polymorphism GG genotype is the most recurrent (Castañeda‐Moreno et al., 2018; Llamas‐Covarrubias et al., 2013), but contrary to expectations, the CC genotype may be considered a lower‐risk factor (OR = 0.18, *p* = 0.02), whereas other authors have reported an association between the C allele and an increased risk of cancer (Vera & Meyer‐Siegler, 2011), especially in prostate cancer (Ding et al., 2009; Razzaghi et al., 2019). However, the differences between our results and those that link the CC genotype to an increased risk of cancer may be due to some factors as different study populations and different types of cancer.

Interestingly, due to the strong linkage disequilibrium among ‐794(CATT)_7_ and ‐173*C alleles, the analysis of this haplotype is more meaningful. Our haplotype frequencies of the analysis of [‐794(CATT)_5–8_/‐173G>C] *MIF* gene polymorphisms exhibited that the 5C (OR = 2.7, *p* = 0.02) and the 7G (OR = 3.39, *p* < 0.01) haplotypes confer a risk factor in SCC. Both haplotypes may lead to an overexpression of the *MIF* gene and a subsequent increase in plasmatic levels which might promote tumor growth and protects cancer cells from immunogenic cell death and other antitumor immune responses (Balogh et al., 2018).

Brocks et al. have found that MIF protects against NMSC by regulating the number of antigen‐presenting cells in the skin and that the loss of keratinocyte‐derived MIF leads to a loss of control of epithelial skin tumor formation (Brocks et al., 2017). However, overexpression of MIF has been found in several human neoplasms, including prostate (Wang, Yao, et al., 2019), breast (Avalos‐Navarro et al., 2019), colorectal (Olsson et al., 2020), endometrial (Xiao et al., 2016), hepatocellular (Zhao et al., 2011), and pancreatic ductal cancer cells (Denz et al., 2010), as well as NMSC (Martin et al., 2009). Utispan et al. have found that MIF promotes proliferation, induces cell cycle progression, and inhibits apoptosis in SCC cell lines (Utispan & Koontongkaew, 2021), while a different group found that MIF promotes invasion and metastasis in oral SCC through the activation of matrix metalloproteins (Wang, Zheng, et al., 2019). Additionally, Heise et al. have detected that normal and immortalized keratinocytes as well as tumor cells from SCC have the capacity to release high amounts of MIF when stimulated by UVB exposure (Heise et al., 2012). According to these studies, we also found a notable more than twice increase in MIF soluble levels (13.93 ng/mL in SCC patients vs 6.00 ng/mL in the RG). This high concentration of MIF in plasm may be promoted by an acute inflammatory response when the skin is exposed to UVB (Martin et al., 2009), and considering SCC's major etiological factor is UV exposure, MIF could play a key role in tumorigenesis and progression of SCC.

Notwithstanding, we previously determined soluble levels of MIF in BCC patients and a different RG, and we found a decreased concentration of MIF in BCC patients (Guevara‐Gutiérrez et al., 2021). These findings may be a clear example that MIF could exhibit a different behavior depending on the mechanisms that have triggered it and that it could have a protective function in acute inflammation but can contribute to carcinogenesis in chronic inflammation as described in SCC. Despite some authors have suggested that MIF may be used as a serological biomarker in some types of non‐cutaneous SCC (Zepeda‐Nuño et al., 2021) or prognostic marker in SCC of the lung (Koh et al., 2019), we did not evaluate the expression of MIF mRNA, nor the expression of MIF in tumoral tissue. These limitations of the work should be taken into account for further work if trying to assess MIF as a biomarker in cutaneous SCC.

Our findings suggest that 5C and 7G [‐794(CATT)_5–8_/‐173G>C] *MIF* gene haplotypes are associated with susceptibility to SCC. We also found that SCC patients exhibit significantly increased MIF soluble levels. However, more research is needed to clarify the impact of *MIF* polymorphisms and haplotypes on the expression of MIF mRNA as well as the mechanism that leads the susceptibility to SCC.

## AUTHOR CONTRIBUTIONS

**Elizabeth Guevara‐Gutiérrez**: manuscript preparation. **Marina Ramos‐Súarez, Romina Angélica Villalobos‐Ayala, and Alberto Tlacuilo‐Parra**: sample collection; **José Francisco Muñoz‐Valle and Victor Tarango‐Martínez**: clinical characteristics determination; **Yeminia Valle, Jorge Ramón Padilla‐Gutiérrez, and José Manuel Rojas‐Díaz**: techniques development and data analysis; **Emmanuel Valdés‐Alvarado**: study design.

## FUNDING INFORMATION

This work was supported by UdG (grant no. PRODEP 2019).

## CONFLICT OF INTEREST STATEMENT

The authors declare that they have no conflict of interest.

## Data Availability

The data that support the findings of this study are available from the corresponding author upon reasonable request.
